# Changes in pubertal hormones, neurotransmitters and sleep patterns during sleep in girls with precocious puberty

**DOI:** 10.1186/1687-9856-2013-S1-P68

**Published:** 2013-10-03

**Authors:** Ersoy Betul, Yazici Pinar, Yilmaz Hikmet, Onur Ece

**Affiliations:** 1 Division of Pediatric Endocrinology, Celal Bayar University, School of Medicine, Manisa, Turkey; 2 Department of Neurology, Celal Bayar University, School of Medicine, Manisa, Turkey; 3 Department of Clinical Biochemistry, Celal Bayar University, School of Medicine, Manisa, Turkey

## Aims

Sleep is one of the essential function for normal physiologic development. During sleep, so many hormonal changes occur. Aim of this study is to determine differences in hormones and neurotransmitters are regarding with onset of puberty during sleep, and sleep pattern, among girls with precocious puberty (PP), premature telarche (PT), and non-pubertal girls.

## Subjects and methods

Thirty nine girls with breast development were underwent full anthropometric and, hormonal assessments. Depending on the results of Gonadotropin-Releasing Hormone (GnRH) test 22 patient, as premature telarche, and the rests, as precocious puberty, were evaluated. Nineteen age-matched, non-pubertal girls, were included as control group. Polysomnography was carried out to all patients. Levels of hormones and neurotransmitters were assessed during REM period.

## Results

Weight Standart Deviation Score (SDS) in girls with PP was significantly higher than those in girls with PT and non-pubertal (p<0.05). Nocturnal kisspeptin, leptin, gamma-amino butyric acid (GABA), and glutamate levels during sleep revealed no statistically significant difference among three groups (p>0.05). First sleep interval of Stage N1 in polysomnography was shorter, whereas total sleep time was longer in girls with premature telarche compared to other groups (p=0,031and p=0.029, respectively).There were negative correlation between total sleep time (TST) and kisspeptin levels and positive correlation between TST and glutamate levels in girls with PT (r=-0.481 and r=0.503, p<0.05, respectively).

## Conclusion

Interval of transition to sleep is shortened, whereas total sleep time is prolonged in girls with premature telarche. Sleep pattern of girls with precocious puberty is similar to those of non-pubertal girls. No change was found serum levels of hormones and neurotransmitters related with pubertal activation during deep sleep periods in girls with precocious puberty compared to girls with PP and non-pubertal.

